# Hypoxia-inducible factor 1 alpha is required for the tumourigenic and aggressive phenotype associated with Rab25 expression in ovarian cancer

**DOI:** 10.18632/oncotarget.7998

**Published:** 2016-03-09

**Authors:** Natividad Gomez-Roman, Neha Mohan Sahasrabudhe, Fiona McGregor, Anthony J. Chalmers, Jim Cassidy, Jane Plumb

**Affiliations:** ^1^ Wolfson Wohl Translational Cancer Research Centre, Institute of Cancer Sciences, University of Glasgow, Glasgow, UK; ^2^ The University Medical Center in Groningen, Groningen, The Netherlands; ^3^ Current address: VP Oncology at Bristol Myers Squibb, Princeton, New Jersey, USA

**Keywords:** Rab25, HIF-1 alpha, tumourigenic, ovarian cancer, intraperitoneal

## Abstract

The small GTPase Rab25 has been functionally linked to tumour progression and aggressiveness in ovarian cancer and promotes invasion in three-dimensional environments. This type of migration has been shown to require the expression of the hypoxia-inducible factor 1 alpha (HIF-1α). In this report we demonstrate that Rab25 regulates HIF-1α protein expression in an oxygen independent manner in a panel of cancer cell lines. Regulation of HIF-1α protein expression by Rab25 did not require transcriptional upregulation, but was dependent on *de novo* protein synthesis through the Erbb2/ERK1/2 and p70S6K/mTOR pathways. Rab25 expression induced HIF-1 transcriptional activity, increased cisplatin resistance, and conferred intraperitoneal growth to the A2780 cell line in immunocompromised mice. Targeting HIF1 activity by silencing HIF-1β re-sensitised cells to cisplatin *in vitro* and reduced tumour formation of A2780-Rab25 expressing cells *in vivo* in a mouse ovarian peritoneal carcinomatosis model. Similar effects on cisplatin resistance *in vitro* and intraperitoneal tumourigenesis *in vivo* were obtained after HIF1b knockdown in the ovarian cancer cell line SKOV3, which expresses endogenous Rab25 and HIF-1α at atmospheric oxygen concentrations. Our results suggest that Rab25 tumourigenic potential and chemoresistance relies on HIF1 activity in aggressive and metastatic ovarian cancer. Targeting HIF-1 activity may potentially be effective either alone or in combination with standard chemotherapy for aggressive metastatic ovarian cancer.

## INTRODUCTION

Ovarian cancer is one of the most lethal types of cancer and is the fifth most common cancer in developed countries [1]. The primary therapeutic regime for ovarian cancer patients involves surgical resection of the tumour followed by platinum based chemotherapy. Although the majority of patients respond to initial treatment, 70% of advanced stage patients will experience relapse, which is typically associated with more aggressive tumours, massive peritoneal dissemination and multi-drug resistance. Although many patients respond to second line treatment with gemcitabine and paclitaxel, there is still no effective therapy for platinum resistant tumours [2]. Thus, the problem of chemoresistance remains a high priority in this disease and the search for novel therapeutic targets continues.

*In vitro* studies and tumour sample analyses have identified a number of genes that associate with enhanced growth and invasiveness of ovarian cancer. One such gene is Rab25, a small GTPase of the Rab11 subfamily involved in endosomal recycling and trafficking pathways [3, 4] that is part of the RAS oncoprotein superfamily. Rab25 expression is upregulated in around 80% of ovarian cancer samples compared to normal ovarian epithelium, and increased Rab25 expression correlates with increasing tumour stage [3]. Enforced Rab25 expression in ovarian cancer cell lines results in increased cell proliferation, inhibition of apoptosis and anoikis and increased aggressiveness *in vivo* [5].

Understanding the Rab25-mediated events that contribute to invasion, migration and metastatic progression could provide new targets for chemotherapeutic intervention.

Rapidly growing tumours outstrip their vascular supply and become hypoxic. Tumour cells that are able to survive in hypoxia exhibit an enhanced propensity to invade [6–8]. In hypoxic conditions, cells adapt to generate energy in oxygen independent ways and minimize cellular damage by inducing the expression of genes involved in angiogenesis, glycolysis, cell survival, invasion, tumour progression and pH regulation, which are mostly regulated by the hypoxia inducible factors (HIFs). HIFs are heterodimers having a constitutively expressed HIF-1β subunit and an oxygen responsive HIF-α subunit [9] which is hydroxylated by prolyl hydroxylase (PHD) enzymes in an oxygen-dependent reaction. This triggers its ubiquitination by the E3 ubiquitin ligase von Hippel-Lindau protein (VHL), which targets HIF-α for 26S proteasomal degradation [10]. In hypoxic conditions, HIF-α escapes degradation, migrates to the nucleus, binds to HIF-1β and stimulates HIF-1 target gene expression [11].

Ovarian cancer generally metastasizes through direct dissemination from the primary site into the peritoneal cavity, without intravasation and extravasation of blood vessels [12]. Elevated levels of nuclear HIF-1α are associated with poor prognosis in ovarian malignancy and have been proposed as independent prognostic biomarkers [13, 14]. Furthermore, HIF-1α protein is overexpressed in the majority of non-hypoxic metastatic tumours [15] and its expression is associated with chemoresistance [16–18]. At present, our understanding of the mechanisms and consequences of HIF-1α induction in non-hypoxic tumours is limited. Rab25 expression in A2780 cells was shown to increase their tumourigenic potential in the peritoneum of immunocompromised mice [19], while SKOV3 cells expressing endogenous Rab25 formed tumours in the peritoneal cavity of nude mice and exhibited elevated levels of HIF-1α expression under non-hypoxic conditions [20]. Based on these observations, we aimed to elucidate the role of HIF-1α in mediating the association between Rab25 expression and the aggressive and tumourigenic phenotype of ovarian cancer cells.

## RESULTS

### Rab25 expression induces HIF-1α expression at atmospheric oxygen concentrations

**Figure 1:**
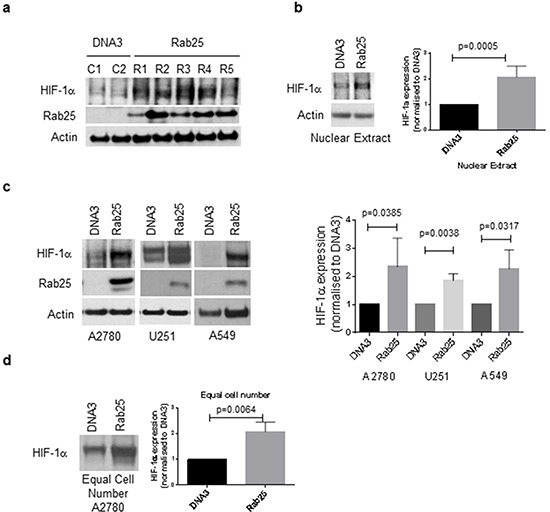
Rab25 induces HIF-1α expression in an oxygen-independent manner **a, b.** Stable cell lines expressing either pcDNA3 (DNA3) or a pcDNA3-Rab25 (Rab25) construct were generated in the ovarian cancer cell line A2780. After selection, the clones were analyzed by Western blot for Rab25 and HIF-1α expression. Clones expressing Rab25 exhibit higher levels of HIF-1α protein, both in cell extracts (a) and nuclear extracts (b). Clones number 1 for Rab25 and 2 for control cells were selected for the remaining experiments. Bars in graph (B) represent means with standard deviation of gels densitometric quantification (ImageJ) normalized to A2780-DNA3 HIF-1α expression of five independent experiments. Statistically significant as analysed by unpaired t-test. **c.** A2780, U251 and A549 cell lines were transiently transfected with 2μg of either pcDNA3 or pcDNA3-Rab25. 48 hours after transfection, protein cell extraction was performed and analyzed by Western blot for HIF-1α and Rab25 expression. Actin is used as loading control. Graph depicts means with standard deviation of gels densitometric quantification (ImageJ) normalized to A2780-DNA3 HIF-1α expression of three independent experiments. Statistically significant as analysed by unpaired t-test. **d.** Western blot analysis for HIF-1α expression from extracts produced using equal number of cells (1×10^7^) of either stable A2780DNA3 or A2780Rab25 cell lines. Graph depicts means with standard deviation of gels densitometric quantification (ImageJ) normalized to A2780-DNA3 HIF-1α expression of three independent experiments. Statistically significant as analysed by unpaired t-test.

To investigate whether Rab25 expression in the ovarian cancer cell line A2780 induces HIF-1α expression, stable cell lines expressing either pcDNA3 (DNA3) or a pcDNA3-Rab25 (Rab25) constructs were generated. After selection, cell extracts of different clones were analysed by Western blot for Rab25 and HIF-1α expression. All clones expressing Rab25 exhibited increased HIF-1α protein levels at atmospheric oxygen concentrations (Figure 1a, Supplementary Figure S1). Having selected Rab25 clone 1 and DNA3 clone 2 for the remainder of the experiments, we demonstrated that nuclear HIF-1α expression was increased in Rab25 expressing cells compared to controls (Figure 1b). To ensure that these effects were not a result of stable cell line generation or specific to ovarian cells, transient transfections were performed in the A2780 cell line as well as the glioblastoma cell line U251 and the lung adenocarcinoma cell line A549. In normoxic conditions, levels of HIF-1α protein were significantly increased after transient transfection of Rab25 in all three cell lines (Figure 1c). These results confirm that the effect of Rab25 on HIF-1α expression is a general phenomenon shared by many cancer cell types and is oxygen-independent.

During these experiments it was noted that Rab25 expression produced a morphological change in A2780 cells, in which an increase in cell diameter was observed (data not shown). To exclude the potentially confounding effect of increased cell volume, cell extracts prepared using equal numbers of either A2780-pcDNA3 or A2780-Rab25 cells were analysed and increased HIF-1α protein expression in Rab25 cell extracts observed as before.

### HIF-1α nuclear localisation and HIF-1 transcriptional activity is observed in Rab25-expressing cells

**Figure 2:**
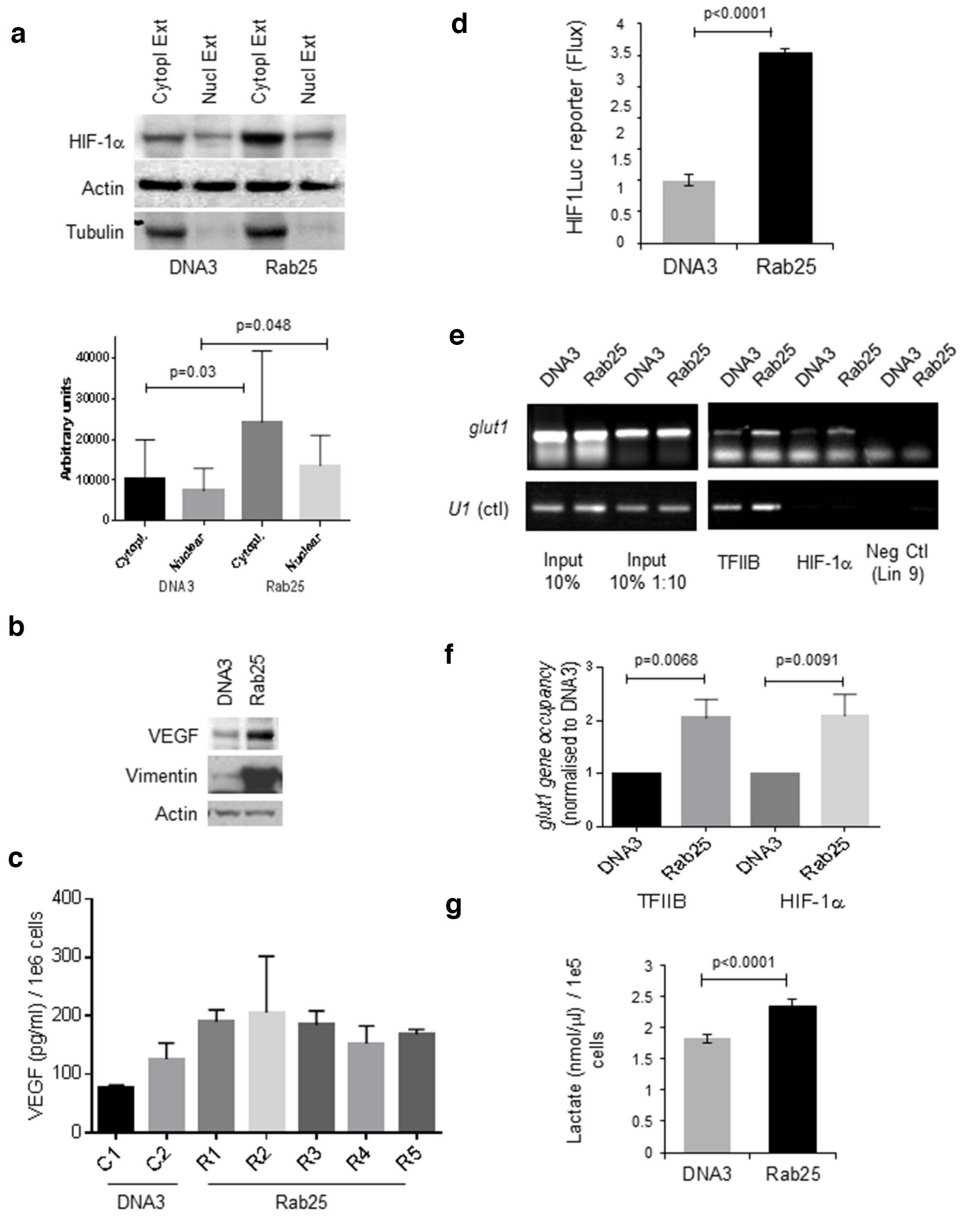
Nuclear localization of HIF-1α and induction of HIF-1 transcriptional activity following Rab25 expression **a.** Cytoplasmic and nuclear extracts (following Dignam protocol [51]) were analyzed by Western blot for HIF-1α expression. Tubulin was used as control for nuclear extract purity. Actin is the loading control. Graph depicts means with standard deviation of gels densitometric quantification (ImageJ) normalized to A2780-DNA3 HIF-1α expression of three independent experiments. Statistically significant as analysed by unpaired t-test. **b.** Whole cell extracts were analyzed by Western blot for VEGF and vimentin expression. Tubulin was used as control for nuclear extract purity. Actin is the loading control. **c.** Determination of secreted VEGF by Elisa assay of media recovered from A2780-DNA3 clones 1 and 2 (C1 and C2) and A2780-Rab25 clones 1 to 5 (C1 to C5). Data presented represents means ± SEM from three independent experiments performed in duplicate. **d.** A2780 cells were transiently transfected with empty vector or Rab25 vector in the presence of a HIF-1 luciferase (HIF1Luc) reporter and analyzed 48 hrs after transfection for luciferase activity using a Xenogen IVIS 50 following addition of luciferin. No differences in cell number were observed following transfection of either pcDNA3 or pcDNA-Rab25 vectors. Data are presented as means ± SEM of triplicates. For statistical significance see Table 1. **e.** Chromatin immunoprecipitation assays performed on A2780-DNA3 or A2780-Rab25 stable cell lines showing increased binding of HIF-1α and the RNA Polymerase II-specific transcription factor TFIIB to the promoter of the HIF-1 target gene Glut-1 in the Rab25-expressing cells compared to control (DNA3). **f.** Graph representing means ± SD of three independent of gels densitometric quantification (ImageJ) normalized to A2780-DNA3 *glut1*. P= statistical significance as analysed by unpaired t-test. **g.** A2780 cells stably expressing either Rab25 or DNA3 were grown for 24 hrs. Media was recovered and tested using BioVision Lactate Assay Kit II, and cells were trypsinized and counted to normalize values against cell number. Lactate concentration is normalized to 1×10^5^ cells per experiment. Data represent mean ± SEM from three independent experiments, each performed in triplicate.

To determine the cellular localisation of HIF-1α in A2780-Rab25 expressing cells, cytoplasmic and nuclear extracts were prepared from A2780-Rab25 cells and analysed by Western blot. While increased HIF-1α protein levels were observed in both cytoplasmic and nuclear fractions of Rab25 cells compared to controls, relative HIF-1α levels were higher in the cytoplasm than the nucleus in both cell populations and nuclear HIF-1α levels were very low in DNA3 cells (Figure 2a). The purity of the fractionated extracts was verified by tubulin immunoblotting.

To investigate HIF-1α transcriptional activity, we analysed the HIF-1 downstream targets vascular endothelial growth factor (VEGF) and vimentin by Western blot. Increased protein levels of both VEGF and vimentin were detected in A2780-Rab25 expressing cells compared to empty vector (Figure 2b). ELISA revealed induction of VEGF to be significantly increased in all Rab25 clones compared with A2780-DNA3 clone 1 and in Rab25 clones 1, 3 and 5 compared with A2780-DNA3 clone 2 (Figure 2c and Table 1 ). HIF-1 luciferase reporter assays provided further confirmation of HIF-1 transcriptional activation by Rab25. In these experiments, A2780 cells were transiently co-transfected with empty vector or Rab25 in the presence of a HIF-1 luciferase reporter vector in which luciferase expression is driven by a HIF-1-dependent hypoxia-response element cloned upstream of a minimal TATA box promoter. A three-fold induction in luciferase activity was observed upon Rab25 expression compared to empty vector (DNA3) under non-hypoxic conditions (Figure 2d). Chromatin immunoprecipitation assays further validated these results, showing significantly increased binding of HIF-1α and the RNA Polymerase II-specific transcription factor TFIIB to the HIF-1 target gene *glut1* promoter in Rab25-expressing cells compared to DNA3 cells (Figures 2e and 2f). This result is specific since HIF-1α binding to the U1 gene promoter, which lacks HIF-1 binding regions, was not observed.

**Table 1:** Increased VEGF levels are observed in Rab25 expressing cells compared to control

VEGF Elisa	p values		p values
C1 vs R1	0.0008	C2 vs R1	0.0051
C1 vs R2	0.0827	C2 vs R2	0.1839
C1 vs R3	0.0017	C2 vs R3	0.0109
C1 vs R4	0.013	C2 vs R4	0.1175
C1 vs R5	<0.0001	C2 vs R5	0.0023

Statistical significance as analysed by unpaired t-test calculated from three independent experiments, each performed in duplicate. C1 and C2 represent A2780-DNA3 clone 1 and A2780-DNA3 clone 2; R1 to R5 represent 5 different A2780-Rab25 clones.

HIF-1 induces the expression of glucose transporters and enzymes involved in glycolysis, increasing the rate of glycolysis in cells and producing excess pyruvate, which is largely converted to lactate [21]. To determine whether Rab25 expression produced such a metabolic shift, we measured lactate production in our stable cell lines. As expected, lactate levels were increased in A2780-Rab25 showed increased levels of lactate compared to control cells (Figure 2g). Overall, our results demonstrate increased HIF-1 activity following Rab25 expression.

### Rab25 expression regulates HIF-1α protein levels through translational activation

**Figure 3:**
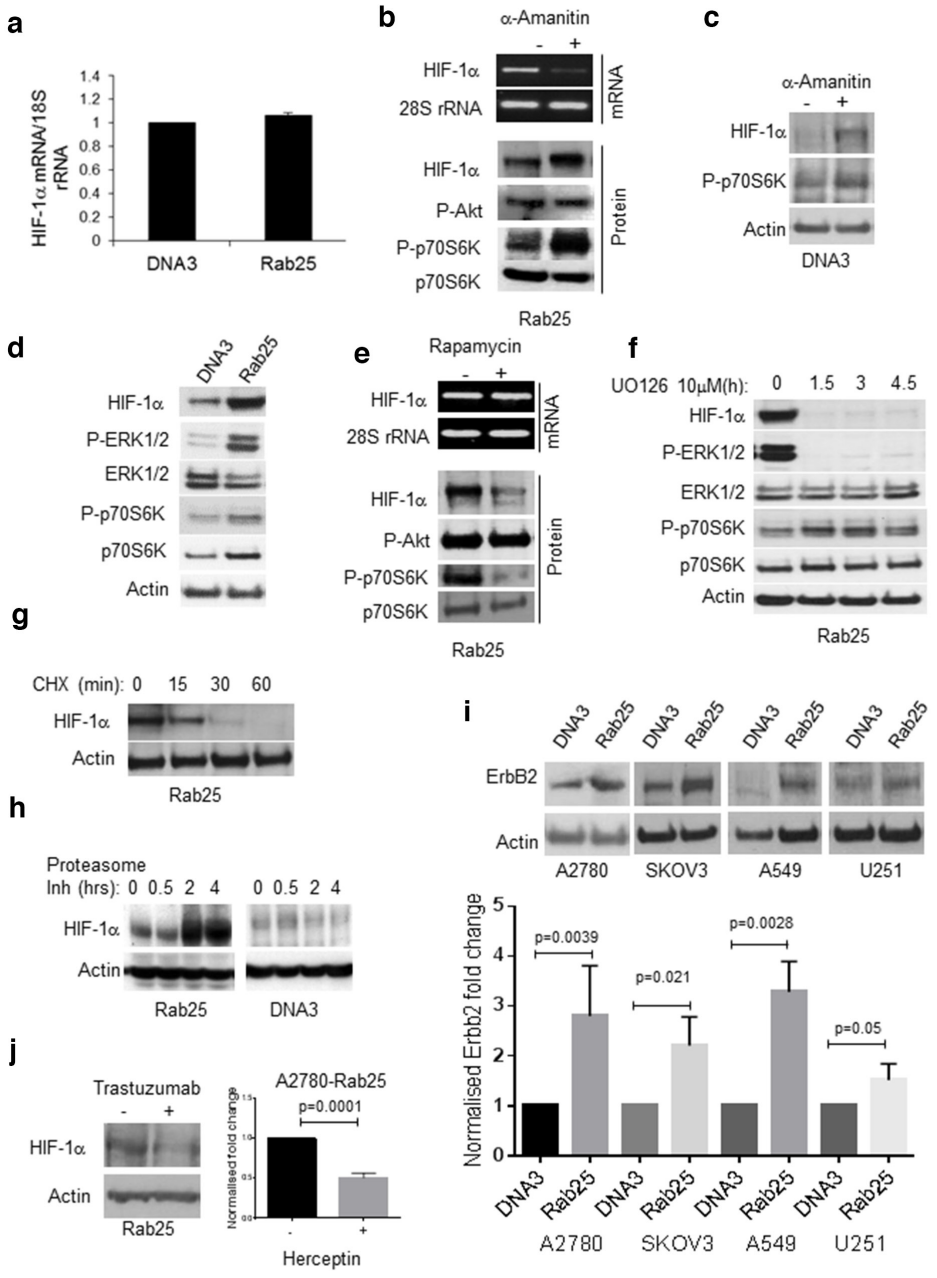
Rab25 induces HIF-1α protein through a translational mechanism **a.** HIF-1α transcript levels were determined by qRT-PCR analysis from mRNA extracts prepared from A2780DNA3 or A2780Rab25 cells. 18S rRNA levels were used as an internal normalization control. Data are presented as means ± SEM of triplicates from three independent experiments. **b, c.** A2780Rab25 cells (b) or A2780-DNA3 (DNA3) cells (c) were treated with either vehicle (DMSO) or a-amanitin (2μg/ml) for 24 hours. mRNA (b and c) and protein (b) were extracted following treatment. mRNA was analyzed by qRT-PCR for HIF-1α (upper panel). 28S rRNA was used as loading control. Protein extracts were analysed by Western blot for HIF-1α, phospho-Akt, phospho- and total-p70S6K. Actin served as loading control. **d.** Western blot analysis of protein extracts prepared from A2780DNA3 and A2780Rab25 stable cells comparing levels of HIF-1α, ERK1/2 (phosphorylated and total), and p70S6K (phosphorylated and total). **e.** mRNA and protein extracts from A2780-Rab25 (Rab25) cells were treated with rapamycin (10nM) for 24 hours and analysed by qRT-PCR and Western blot as in B. **f.** A2780Rab25 stable cells were treated with the MEK inhibitor U0126 (10μM) and protein extracts were prepared at different time points and analysed by Western blot. **g.** Protein translation was inhibited by cycloheximide (100μg/ml) and protein extracts were prepared at the indicated time points and analyzed by immunoblotting. **h.** Protein degradation was inhibited by MG-132 (1 μg/ml) and protein extracts were prepared at the indicated time points and analyzed by immunoblotting. **i.** Analysis of Erbb2 expression from a panel of cell lines: A2780DNA and A2780Rab25 cell lines and SKOV3, A549 and U251 cells transiently transfected with pcDNA3 or pcDNA3-Rab25. Graph depicts means with standard deviation of gels densitometric quantification (ImageJ) normalized to A2780-DNA3 HIF-1α expression of three independent experiments. Statistically significant as analysed by unpaired t-test. **j.** HIF-1α expression analysis in A2780Rab25 cells following Herceptin treatment for 24 hours.

HIF-1α expression can be regulated transcriptionally, translationally and/or through post-translational events (e.g. protein stabilisation). Similar levels of HIF-1α mRNA were detected in A2780-Rab25 and DNA3 cells, demonstrating no transcriptional upregulation (Figure 3a). To confirm these observations, cells were treated with a-amanitin at a concentration which specifically blocks transcription of RNA polymerase II (2mgml^−1^). In Rab25 cells, mRNA levels of HIF-1α declined after a-amanitin treatment whereas the 18S rRNA transcript transcribed by RNA Polymerase I remained at a steady level and an increase in HIF-1α protein was observed (Figure 3b). This effect on HIF-1α was replicated in the low expressing A2780-DNA3 cells, in which a-amanitin treatment induced HIF-1α protein expression (Figure 3c). Consistent with previous reports that transcription inhibitors such as a-amanitin activate the mTOR signalling pathway [22], activation of the downstream mTOR target p70S6K following a-amanitin treatment was detected in our cell lines (Figures 3b and 3c). These data indicate that activation of the mTOR/p70S6K pathway might be sufficient to enhance HIF-1α protein levels in the A2780 cell line at normal oxygen concentrations. Because Rab25 can activate mTOR/p70S6K [23], we compared activation of p70S6K in A2780 cells expressing either Rab25 or empty vector (DNA3). Increased p70S6K phosphorylation was observed in Rab25 cells compared to controls (Figure 3d), and this was suppressed following mTOR inhibition with rapamycin (Figure 3e). Selective inhibition of mTOR also resulted in a reduction of HIF-1α protein levels in A2780-Rab25 overexpressing cells (Figure 3e), confirming our previous observations. Taken together, our results demonstrate a role for the mTOR/p70S6K pathway in mediating Rab25 regulation of HIF-1α expression.

ERK1/2 signalling has also been implicated in the translational and post-translational control of HIF-1α [24–26], through inhibition of protein degradation in an oxygen-independent manner [27]. Consistent with this, increased ERK1/2 activation was observed in Rab25 cells compared to control (Figure 3d). To determine if ERK is involved in Rab25 mediated induction of HIF-1α, protein extracts from cells treated with the specific MEK inhibitor UO126 were analysed by Western blot. U0126 treatment of A2780-Rab25 cells resulted in complete suppression of ERK1/2 phosphorylation and a corresponding reduction of HIF-1α protein to almost undetectable levels (Figure 3f). Similar effects on HIF-1α levels were obtained using the MEK inhibitor PD98059, although to a lesser extent than UO126 (data not shown). While crosstalk between ERK signalling and p70S6K has been previously reported [28], we did not observe any changes in p70S6K phosphorylation after ERK inhibition (Figure 3f, lower panels), indicating that HIF-1α regulation by the ERK pathway is mTOR/p70S6K-independent.

ERK1/2 can also regulate HIF-1α post-translationally by inhibition of ubiquitin-dependent proteasomal degradation [29–32]. To assess post-translational regulation of HIF-1α by Rab25, the kinetics of HIF-1α protein decay were investigated following treatment with the protein synthesis inhibitor cycloheximide (CHX). Treatment with CHX resulted in a 50% reduction of HIF-1α protein levels after 15 min and complete disappearance after 60 min (Figure 3g). To confirm a translational induction of HIF-1α by Rab25, we hypothesized that inhibition of protein degradation would cause HIF-1α accumulation only in Rab25 cells, with little or no effect on DNA3 control cells. Consistent with this hypothesis, treatment with the proteasome inhibitor MG-132 (1 μM) resulted in accumulation of HIF-1α in Rab25 cells as predicted, whereas no increase in HIF-1α was detected in DNA3 cells (Figure 3h). These results indicate that Rab25 expression does not control the degradation and protein stability HIF-1α, but induces its translation.

The ERK and mTOR/p70S6K signalling pathways respond to activation of a variety of tyrosine kinase receptors one of which is ErbB2 [33–35]. In breast cancer patients, amplification and/or overexpression of ErbB2 is strongly associated with worse prognosis and a higher incidence of metastases [36, 37]. Expression of Rab25 was associated with significant increases in ErbB2 protein levels in a panel of difference cancer cell lines including A2780, SKOV3, A549 and U251 cell lines (Figure 3i). The observation that HIF-1α protein levels were significantly reduced in Rab25 cells treated with trastuzumab, a monoclonal antibody targeting ErbB2, indicates that HIF-1α induction by Rab25 appears to be regulated by ErbB2 as well as Erk1/2 and mTOR signalling pathways.

### Rab25-induced epithelial to mesenchymal phenotype requires HIF-1 activity

**Figure 4:**
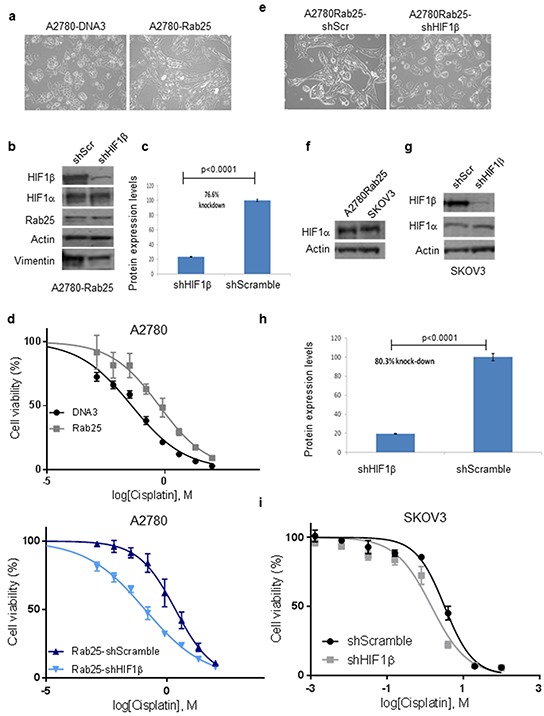
Epithelial to mesenchymal transition and cisplatin resistance is HIF-1 dependent **a.** Representative bright field images of A2780-DNA3 and A2780-Rab25 cells in culture. **b.** Cell extracts prepared from A2780Rab25-shScr or A2780Rab25-shHIF1β stable cell lines were analysed for protein expression levels for HIF-1β, HIF-1α, vimentin and Rab25 by Western blot. Actin served as loading control. HIF-1β expression levels were calculated from three independent experiments and percentage knockdown was calculated using densitometric quantification using densitometric quantification. The fraction of the average is presented. **c.** Graph representing means with standard deviation of gels densitometric quantification (ImageJ) normalized to A2780-shScramble HIF-1β expression of three independent experiments. P= statistical significance as analysed by unpaired t-test. **d.** Cell viability assays following cisplatin treatment at a concentration range. Means of three independent experiments for each cell line are presented (error bars represent ±SEM). **e.** Representative bright field images of A2780Rab25-shHIF1β and A2780Rab25-shScr cells. For the generation of A2780-Rab25 cells expressing shRNAs, cells were transduced with a lentiviral construct expressing either shScramble (shScr) or shHIF-1β RNA and selected with puromycin. **f.** Comparison of HIF-1α protein expression in A2780-Rab25 and SKOV3 cell lines by Western blot. **g.** HIF-1β expression levels in SKOV3-shHIF1β and SKOV3-shScr cell lines, along with unchanged HIF-1α expression levels and actin as loading control. **h.** Each sample in G was repeated thrice and percentage knock-down was calculated using densitometric quantification. **i.** Cell viability assays following cisplatin treatment at a concentration range. Means of three independent experiments for each cell line are presented (error bars represent ±SEM).

VHL-silenced renal carcinoma cells take on an elongated, fibroblastoid morphology while VHL(+) cells grow as clusters of cuboidal and rhomboid cells. This phenotypical change from cuboidal (epithelial) to fibroblastoid (mesenchymal) morphology has been termed ‘epithelial to mesenchymal transition’, and requires HIF-1α activation [38]. Expression of Rab25 in intestinal cells triggers the same EMT phenotype as VHL-silenced cells [39]. We observed a similar effect of Rab25 expression on cellular morphology of A2780 cells with flat, cuboidal DNA3 cells adopting an elongated, fibroblastoid morphology when expressing Rab25 (Figure 4a). In order to interrogate the importance of HIF1 activity on the phenotypical changes associated with Rab25 expression, A2780Rab25 cells were transduced with a lentivirus construct containing a short-hairpin (sh) RNA targeting HIF1β. Trasduction with this lentiviral construct has previously been demonstrated to stably inhibit HIF-1 transcriptional activity [40]. By targeting the ubiquitously expressed HIF-1β subunit, HIF1 activity is inhibited irrespective of oxygen conditions and/or HIF-a subunit expression, making this approach a more robust method by which to abolish HIF-regulated gene expression. Following stable cell line selection, A2780Rab25-shHIF1β cells exhibited a statistically significant 76.6% reduction in HIF-1β protein expression compared to A2780-Rab25 cells stably expressing scrambled shRNAs (A2780Rab25shScr, Figure 4b and 4c). HIF1α expression levels remained unchanged in both cell lines (Figure 4b). Whereas the elongated phenotype persisted in A2780-Rab25shScr cells, HIF-1β knockdown caused this elongated morphology to revert to the cuboidal phenotype of the parental cells (Figure 4e). Consistent with previous observations that vimentin expression is associated with EMT, as well as being a HIF-1 target gene [41], HIF-1β depletion was shown to reduce vimentin expression in A2780-Rab25-shHIF-1β compared to control (Scr) cells (Figure 4b). These data provide evidence that HIF-1 transcriptional activity regulates the EMT program in the ovarian cancer cell line A2780 following Rab25 expression.

### Rab25 induced cisplatin resistance is HIF-1-dependent

Rab25 was initially identified in a series of ovarian cancers that did not respond to chemotherapy [5] and subsequently shown to prevent apoptosis and anoikis induced by taxol-based chemotherapy. In line with these observations, Rab25 expression significantly increased cisplatin resistance of A2780 cells compared to controls (p=0.0014, calculated by Two-way ANOVA), as measured by cell viability assay (Table 2 and Figure 4d).

**Table 2:** In vitro cytotoxicity of free cisplatin in the human ovarian cancer cell line A2780 expressing empty vector (DNA3), Rab25, Rab25 and scramble short-hairpin (shScr) or Rab25 and shHIF1β

IC_50_ for Cisplatin (95%CI), μM	
A2780DNA3	0.0387 (0.03006-0.05004)
A2780Rab25	0.5737 (0.3944-0.8344)
A2780Rab25-shScr	2.223 (1.782-2.772)
A2780Rab25-shHIF1β	0.1235 (0.1043-0.1463)
SKOV3-shScr	3.49 (2.677-3.879)
SKOV3-shHIF1β	1.65 (1.111-1.915)

As assessed by cell viability assay (MTT). IC50 calculated from three independent experiments.

HIF-1α expression is also known to induce chemoresistance in cancer cells. To assess the role of HIF-1 activity in the resistance conferred by Rab25 expression, cisplatin sensitivity was determined by MTT assay in Rab25shHIF-1β, shScramble-Rab25 and SKOV3 ovarian cancer cells. SKOV3 cells, which express similar endogenous levels of HIF-1α to A2780-Rab25 overexpressing cells (Figure 4f), were transduced as previously described with a lentivirus encoding shHIF-1β to generate stable cell lines in which HIF-1β protein expression was reduced by 80.3% (Figures 4g and 4h). Consistent with our previous findings, HIF-1α expression levels were unchanged after knock-down of HIF-1β (Figure 4g). HIF-1β knock-down resulted in statistically significant sensitisation of A2780-Rab25 and SKOV3 cells to cisplatin compared to shScramble control cells (p=0.0014 for A2780 Rab25-shScramble vs A2780 Rab25-shHIF1b, and p=0.009 for SKOV3-shScramble vs SKOV3-shHIF1b, as calculated by 2way ANOVA), with ∼50% reduction in cisplatin IC50 values (Figure 4d and i, Table 2 ). HIF-1β knock-down reversed the cisplatin resistance conferred by Rab25 expression in A2780 cells to similar levels observed in A2780-DNA3 cells, (Figure 4d and Table 2). These results indicate that the chemoresistance imposed by Rab25 expression correlates with HIF-1 activation.

### Inhibition of HIF-1 activity reduces Rab25-associated intraperitoneal tumourigenicity of ovarian cancer cells

**Figure 5:**
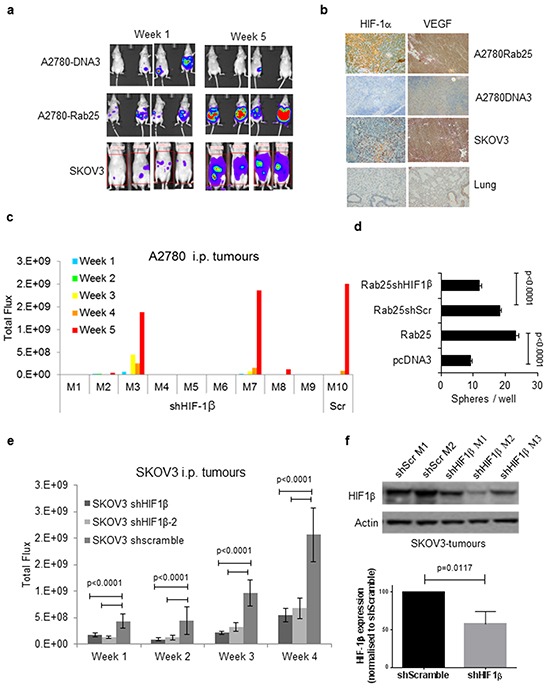
In vivo tumorigenic potential of A2780-Rab25 and SKOV3 cells is HIF1-dependent **a.** Representative images of mice injected i.p. with 5×10^6^ of A2780, A2780-Rab25 or SKOV3 cells stably expressing luciferase and monitored over time by measuring luciferase expression after s.c. administration of luciferin (30mg/ml) using an IVIS-50 imaging system. **b.** HIF-1α and VEGF immunohistochemistry images of lung, intraperitoneal (i.p.) or subcutaneous (s.c.) tumours taken from mice at 28 days after inoculation. **c.** Longitudinal studies of i.p. tumours from mice injected with 5×10^6^ of A2780Rab25-shHIF1β or A2780Rab25-shScr cells. Mice were monitored once a week for five weeks by bioluminescence. **d.** Sphere formation assay in A2780-DNA3, A2780-Rab25, A2780-Rab25shScr and A2780-Rab25shHIF-1β. Each experiment was repeated thrice and error bars represent SEM; *p<0.005. **e.** Longitudinal studies of i.p. tumours from mice injected i.p. with SKOV3-shHIF1β1 and SKOV3-shHIF1β2 compared to SKOV3-shscramble-Lluc. Each cell line was intraperitoneally injected into six nude mice and tumours were monitored for 4 weeks through luminescence (Total flux). Graph depicts means of six mice per cohort, error bars represent SEM. Statistical significance calculated for each SKOV3-shHIF-1β vs control cell line SKOV3-shScramble using two-way ANOVA. **f.** Western blot for HIF1β expression in tumour samples obtained from three mice (M1 to M3) injected with either SKOV3-shHIF1β1 (shHIF1β) or SKOV3-shScr cells (shScr) for 4 weeks. Actin served as loading control. Graph representing HIF1β knock-down normalised protein levels obtained by densitometric quantification of bands from extracts of three independent SKOV3-shHIF-1β tumours and compared HIF1β levels from SKOV3-shScramble tumours. Three independent SKOV3-shScramble tumours were analysed and averaged.

To assess the tumourigenic potential of our cell lines, an ovarian peritoneal carcinomatosis mouse model was developed by injecting either A2780-Rab25 or A2780-DNA3 cells in the peritoneal cavity of athymic nude mice (MF1 nu/nu). Tumour growth was monitored by non-invasive bioluminescent imaging after intraperitoneal injection of cells stably expressing firefly luciferase. As previously reported [5], 100% of the mice injected with A2780-Rab25 cells developed peritoneal disease compared to 25% of mice injected with A2780-DNA3 cells (Figure 5a and Table 3). In comparison, no difference in tumorigenicity was observed in two cohorts (n = 6 mice) injected subcutaneously with either Rab25 or DNA3 expressing cells, (data not shown). Since Rab25 intraperitoneal xenografts harvested five weeks after inculation exhibited higher levels of HIF-1α and VEGF expression than A2780-DNA3 tumours (Figure 5b), the effect of HIF-1 activity on tumorigenicity and invasiveness was investigated by injecting luciferase expressing A2780Rab25-shHIF-1β and SKOV3-shHIF1β cells intraperitoneally as described above. Luminescence was detected in four out of nine mice in the A2780Rab25-shHIF1β cohort at week 5 (Figure 5c and Table 3 ), in two of which the signal was comparable to that of A2780-Rab25 and A2780-Rab25shScr tumours. In the remaining two mice, luminescence was weak at week 5, and no visible tumour was apparent on internal examination of the peritoneum (Figure 5c, Table 3 ). In contrast, strong luminescence was detected in all mice injected with A2780Rab25-shScr cells, and tumours disseminated throughout the peritoneal cavity were observed on internal examination (Figure 5c and Table 3 ).

**Table 3:** Tumorigenic potential of ovarian cancer cell lines A2780 and SKOV3 either scramble (Scr) or HIF-1β short hairpin RNAs

Cell line	Tumour Incidence	Percentage
A2780DNA3	2/8	25%
A2780Rab25	8/8	100%
A2780Rab25shScr1	8/8	100%
A2780Rab25shScr2	7/8	87%
A2780Rab25shHIF1β	2/9	22.22%
SKOV3	6/6	100%
SKOV3-shScr	6/6	100%
SKOV3-shHIF1β	6/6	100%

Mice were injected intraperitoneally with 5×10^6^ cells and monitored for signs of disease (abdominal inflammation, weight loss). Experiment endpoint was at six weeks.

Recently, HIF-1α has been shown to be essential for the maintenance of ‘tumour initiating’ or ‘cancer stem cells’ (CSCs) in various cancer types [42–44]. Based on our previous data, which support this hypothesis in the context of ovarian cancer, tumour sphere assays were performed using the panel of ovarian cancer cell lines described above. In this assay, tumour spheres are grown in anchorage-independent and serum-free conditions can be used as an *in vitro* surrogate of tumourigenicity. The resulting spheres are enriched in cancer stem-like cells and are capable of serial propagation of their original tumour phenotype in animals [45]. Rab25 expressing cells formed significantly more spheres than the control cell line A2780-DNA3 (Figure 5d), and although a modest decrease in sphere-forming efficiency was observed in cells transduced with shScramble lentivirus, which might be attributable to the dual antibiotic selection required, HIF-1β knock-down significantly reduced the number of spheres formed compared to either Rab25shScramble or Rab25 expressing cells (Figure 5d). Taken together, our results demonstrate that HIF-1 activity is required for both maintenance of CSC phenotype in A2780 cells and their tumourigenicity *in vivo*.

To exclude the possibility that the HIF-1 requirement for A2780-Rab25 tumourigenicity is specific to this cell line, a second ovarian peritoneal mouse model was developed using the SKOV3 cell line, which expresses endogenous Rab25 and HIF-1α at atmospheric oxygen concentrations. 100% of mice injected with SKOV3 cells developed tumours in the peritoneal cavity (Table 3). In order to investigate HIF-1 dependency of SKOV3 tumourigenicity, three cohorts of mice were injected with either SKOV3-shHIF-1β clone 1 (SKOV3-shHIF-1β), SKOV3-shHIF-1β clone 2 (SKOV3-shHIF-1β-2) or SKOV3-shScr. Although HIF-1β knock-down did not reduce SKOV3 tumourigenicity (Table 3), it had an impact on tumour burden as indicated by the significantly reduced luminescent signal obtained from SKOV3-shHIF-1 tumours compared with those derived from SKOV3-shScr cells (Figure 5e). Protein was extracted from tumours formed by SKOV3-shHIF1β and SKOV3-shScr and analysed for HIF-1β expression by Western blot. The percentage knock-down seen in SKOV3-shHIF-1β tumours (56.1%, 75.04% and 41.08%, Figure 5f) was lower than that seen in cells grown in culture for the same period of time (80.6% knock down, Figure 4e). This re-expression of HIF-1β *in vivo* might explain why there was no change in tumourigenicity.

## DISCUSSION

This study aimed to determine the role of HIF-1 in the invasive and metastatic ovarian cancer phenotype associated with expression of the small GTPase protein Rab25. Induction of HIF-1α was observed upon expression of Rab25 across a panel of cancer cell lines of different origins. HIF-1α induction occurred in parallel with a variety of tumour responses including EMT, metabolic reprogramming, chemoresistance and was strongly associated with increased peritoneal tumourigenicity in nude mice. All these effects were reversed upon HIF-1 transcriptional inactivation through HIF-1β knock-down. Rab25 increased HIF-1α expression through an Erbb2 and ERK1/2 and p70S6K/mTOR dependent mechanism in an oxygen-independent manner. To our knowledge, this study is the first to show upregulation of HIF-1α via ErbB2. Rab GTPases are critical for mediating signalling transduction propagation from growth factors via the regulation of their intracellular localisation. A role for Rab25 in regulating epidermal growth factor receptor (EGFR) trafficking has been previously reported [46]. Our novel observation that Rab25 might also be involved in Erbb2 regulation warrants further investigation.

It has been suggested that Rab25 promotes the cancer pathophysiological phenotype by regulating cellular ATP levels and glycogen stores that protect cancer cells from bioenergetic stress. For example, Rab25 has been shown to increase glucose uptake by binding and trafficking the HIF-1 target gene GLUT1 to the cytoplasmic membrane [47]. We report here increased binding of HIF-1 to the GLUT1 promoter following Rab25 expression. These data suggest that Rab25 is not only involved in regulating cellular localisation of GLUT1 but also its abundance at the transcriptional level. Such an effect on both cellular localisation and transcriptional control of GLUT1 by Rab25 has the ability to ensure cancer cell survival under nutrient stress conditions. HIF-1 activation also results in a large increase in glycogen stores [48]. Increased glycogen accumulation in Rab25-expressing cells has been observed both *in vitro* and *in vivo* in ovarian cancer patient tumour samples [48]. HIF-1 activation provides an additional mechanism by which Rab25 could produce glycogen accumulation. Our results help explain why these cells can metastasize and invade the peritoneum during acute bioenergetics stress. In addition, several HIF-1 targets can regulate peritoneal invasion *in vivo.* These include VEGF, MMP1 (matrix metallopeptidase 1) and 9, TGF-α (transforming growth factor α) and/or IGF-2 (insulin-like growth factor-2) [49]. The models that we have developed might help elucidate what target genes are essential for ovarian peritoneal carcinomatosis, allowing identification of novel therapeutic targets.

In conclusion, our results demonstrate that HIF1 is a major factor controlling the aggressive and chemotherapy-resistant phenotype of Rab25 expressing ovarian cancers and indicate that targeting HIF-1 activity might be effective either alone or in combination with standard chemotherapy for aggressive metastatic ovarian cancer.

## MATERIALS AND METHODS

### Materials

Cisplatin, MTT [3-(4,5-dimethylthiazol-2-yl)-2,5-diphenyltetrazolium bromide] were obtained from Sigma-Aldrich Co. Ltd (Dorset, UK). Luciferin was obtained from Caliper Lifesciences Ltd (Wellfield, UK). G418S Sulphate solution was obtained from ForMedium Ltd (UK). Blasticidin, Lipofectamine 2000, Virapower Promoterless Lentiviral Gateway Kit were obtained from Invitrogen Co. (Paisley, UK). Lactate was measured using BioVision Lactate Assay Kit II. VEGF was quantified by VEGF Elisa from Calbiochem. The following antibodies were used: Actin from Santa Cruz Biotechnology; Bak and Bax from Cell Signalling Technology; HIF-1α, HIF-1β and PARP from BD Transduction Laboratories; p53 from Leica Microsystems; and vimentin from Abcam.

### Luciferase reporters

The luciferase reporter was constructed using the ViraPower Promoterless Lentiviral Gateway Expression System according to manufacturers' instructions. It contains the luciferase gene controlled by the Ubiquitin C promoter. The luciferase gene was amplified from plasmid pGL4-CMVLuc (Promega) using the following primers: Forward-5′ CACCATGGAAGACGCCAAA; Reverse-5′ AAACACGGCGATCTTTCCG. The p53 reporter was amplified from p53 Luciferase Reporter Vector (Panomics) using the following primers: Forward-5′ GGTACCGAGCTCTTA; Reverse-5′ GCT TTACCAACAC. All PCR reactions were carried out with Proofstart DNA Polymerase (Qiagen) following manufacturer's protocols. Cloning of the luciferase product into pENTR^TM^-gene was done according to manufacturers' instruction (Virapower^TM^ Promoterless Lentiviral Gateway® Kits). Cloning product was confirmed by sequencing with ABI PRISM 3130XL Genetic Analyzer (Applied Biosystems, Foster City, CA). Lentiviral construct was performed by recombination of the supplied plasmids pENTR^TM^5′/UbCp, containing the ubiquitin C promoter, pLenti6/R4R2/V5-DEST and the cloned pENTR^TM^-luciferase. Transfection was performed as described previously in HEK293T cells. Virus was harvested 72hr post transfection, filtered through 0.45μm filter (Millipore, Bedford, MA) and resuspended in fresh medium or array buffer. Titers of lentiviral preparations were determined using A2780 cells and were around 10^6^ IFU/ml. To infect cells using lentiviral vectors, cells were placed in a 6-well plate at a density of 5×10^7^ million cells/well the day before infection. The next day medium was removed and 2ml lentivirus in cell culture medium was added in the presence of 2μg/ml polybrene to initiate infection. Viruses were removed 24hr after infection and fresh cell culture medium was added. After 24 hr, blasticidin (2.5mg/ml) was added and selection performed for cells expressing luciferase.

### Cell lines

The human ovarian carcinoma cell line A2780 was obtained from Dr. R. F. Ozols (Fox Chase Cancer Centre, Pennsylvania, U.S.A.). The SKOV3 cell line was obtained from ATCC. Cells were maintained in Roswell Park Memorial Institute 1640 medium (Invitrogen) containing glutamine (2mM) and foetal calf serum (10%). The stable cell lines expressing Rab25 or empty vector were generated by selection of transfected cells with pcDNA3-Rab25 or pcDNA3 plasmid using Lipofectamine 2000 according to manufacturers' instructions. Once stables cells were selected and analysed for Rab25 expression, they were transduced with a firefly luciferase reporter previously generated and clones were selected with blasticidin. Stable cell lines expressing Rab25 and luciferase were grown in the presence of geneticin (0.5mg/ml) and blasticidin (2.5mg/ml). Cells were grown in the absence of drugs for 24 hours before in vivo experiments and for the duration of experiment for in vitro assays.

shRNA cloned in pLKO.1 vector (Reference code: TRCN0000003819 the TRC library, Open Biosystems, now under the Thermoscientific umbrella and the reference is http://www.broadinstitute.org/rnai/public/clone/details?cloneId=TRCN0000003819) targeting HIF1β was used for knockdown in A2780Rab25-Lluc and SKOV3-Lluc using lentiviral gene delivery method. Lentivirus construct was made using Virapower promoterless Lentiviral Gateway kits (Invitrogen^TM^) according to manufacturer's instructions using pLKO.1 vector with shHIF1β as the pLenti-based expression plasmid. Non-targeting shRNA scramble was used as negative control. pLKO.1 plasmid has Puromycin resistance gene and RPMI with puromycin was used for selecting successfully transduced cells.

### Protein extraction from tissue

Tissue was ground into a fine powder in liquid nitrogen and lysis buffer (0.5%NP40 (Sigma^®^), 250nM Sodium Chloride and 50mM HEPES pH7.0) supplemented with PIC (Roche) in 5:1 proportion was added to powder; and was incubated at room temperature for 15 min. Protein extract was obtained by centrifugation at 16100g for 10min.

### Cytotoxicity assay

Drug sensitivity was determined by tetrazolium based chemosensitivity assay as described previously [50]. Briefly, cells were plated out at a density of 1×10^3^ per well in 96-well flat bottomed plates (IWAKI) and allowed to attach and grow for 2-3 days. They were exposed to cisplatin for 24 hr and then fed with fresh medium daily for 3 days. On the fourth day, cells were fed with medium containing HEPES buffer (10mM) and MTT (50mL, 5mg/mL) was added to each well. Plates were incubated in the dark at 37°C for 4 hr, medium and MTT removed and the MTT-formazan crystals dissolved in dimethyl sulphoxide (200mL/well). Glycine buffer (25mL/well, 0.1M, pH 10.5) was added and the absorbance measured at 570nm in a multi-well plate reader (Model *Emax*, Molecular Devices Ltd., Wokingham UK).

For drug addition a serial dilution of eight concentrations of cisplatin was prepared. Four wells were used for each drug concentration. Cisplatin was solubilised in dimethyl sulphoxide before addition to medium with a final concentration of less than 1%.

Results are expressed in terms of the drug concentration required to kill 50% of the cells (IC_50_) estimated as the absorbance value equal to 50% of that of the control untreated wells.

### Tumour growth *in vivo*

Cell lines A2780 and A2780-Rab25 were established as xenografts in six to eight-week-old athymic female nude (MF1 nu/nu) mice. Monolayer cultures were harvested with trypsin/EDTA and resuspended in PBS. Animals were housed in autoclaved microisolator cages in an air-filtered laminar flow cabinet and were given food and water ad libitum. All procedures were performed under sterile conditions in a laminar flow hood. This animal experiment was in compliance with all regulatory guidelines.

For luminescence recording, mice were injected s.c. with 200ml of the D-luciferin substrate (30mg/ml). Following injection, mice were anaesthetised with isoflurane (3% and 1 litre/min oxygen) and placed in the imaging system. Luminescence was recorded 10 min after luciferin injection with an exposure time of 1 min using the Xenogen IVIS50 imaging system from Caliper Life Sciences Ltd. (Wellfield, UK). Imaging was repeated weekly to allow estimation of tumour burden over time.

For i.p. xenografts, about 5×10^6^ cells were injected into the peritoneal cavity of mice and mice were imaged on a weekly basis.

Analysis of the bioluminescent images was performed by defining a set region of interest to be used in all animals. For the disseminated model, the signal over the entire abdomen of the mouse was analysed.

### Tumour sphere forming assay

Tumour sphere forming assay was performed as previously described with few modifications [44]. Cells were seeded at 100 cells per well in a 96-well plate and left for 7 days, replenishing with 25ml of serum-free medium every other day. Serum-free medium consisted of Ad/DMEM/F12 (Invitrogen, Paisley UK) supplemented with 10 U/ml heparin, 2% B27 (Invitrogen), human recombinant fibroblast growth factor 2 (FGF-2, 20 ng/ml, Sigma-Aldrich) and epidermal growth factor (EGF, 20 ng/ml, Sigma-Aldrich). After 7 days, properly formed spheres were counted. The data calculated for the number of spheres is the average of three independent experiments. Spheres were counted from 32 different wells/cell line/experiment.

### Statistical analysis

Experiment results obtained were statistically evaluated by simple t-test. Differences were considered significant if P<0.05. Statistical analysis was performed by SigmaPlot 10.0.

## SUPPLEMENTARY FIGURE


