# Supplementary material for: Hypoxia-inducible factor 1 alpha is required for the tumourigenic and aggressive phenotype associated with Rab25 expression in ovarian cancer
Source: Oncotarget. 2016 Mar 9;7(16):22650–64. doi: 10.18632/oncotarget.7998 (PMC5008389; doi:10.18632/oncotarget.7998)
Supplement: Supplementary file 1 [file oncotarget-07-22650-s001.pdf]

SUPPLEMENTARY FIGURE

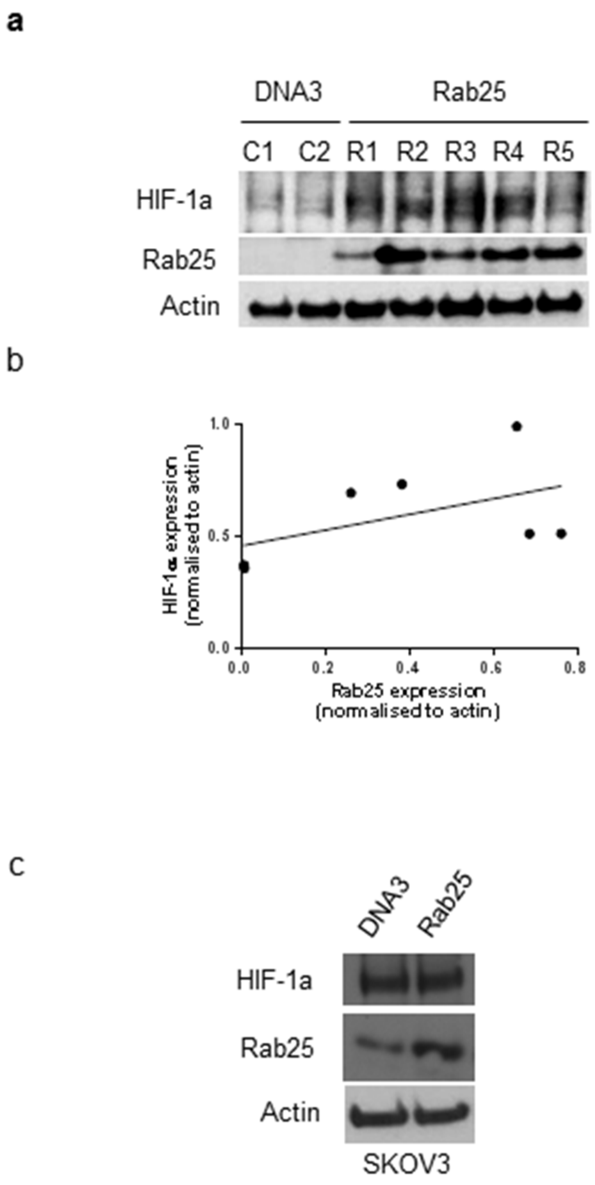

Supplementary Figure S1: Rab25 induces HIF-1α expression in an oxygen-independent manner. a. Stable cell lines expressing either pcDNA3 (DNA3) or a pcDNA3-Rab25 (Rab25) construct were generated in the ovarian cancer cell line A2780. After selection, the clones were analyzed by Western blot for Rab25 and HIF-1α expression. **b.** Plot graph representing the correlation between Rab25 and HIF-1α expression in the different cell clones. Gel densitometric quantification of HIF-1a and Rab25 (normalised to actin) was performed using ImageJ software. **c.** Transient transfection of either pcDNA3 (DNA3) or a pcDNA3-Rab25 (Rab25) in the ovarian cancer cell line SKOV3. Protein extraction was performed 48 hours post-transfection and analysed by Western blot.
